# Editorial: Lectins and Their Ligands in Shaping Immune Responses

**DOI:** 10.3389/fimmu.2019.02379

**Published:** 2019-10-09

**Authors:** Bernd Lepenies, Roland Lang

**Affiliations:** ^1^Immunology Unit and Research Center for Emerging Infections and Zoonoses, University of Veterinary Medicine, Hanover, Germany; ^2^Institute of Clinical Microbiology, Immunology and Hygiene, Universitätsklinikum Erlangen, Friedrich-Alexander-Universität Erlangen-Nürnberg, Erlangen, Germany

**Keywords:** glycoimmunology, lectins, C-type lectins, galectins, siglecs, myeloid cells, dendritic cells, immunomodulation

Lectins are glycan-binding proteins that are involved in numerous biological processes including cell development, cell–cell interactions, signaling pathways, and the immune response. In innate immunity, lectins often act as pattern recognition receptors (PRRs) and recognize pathogen-associated molecular patterns (PAMPs), but also damage-associated molecular patterns (DAMPs). Thus, lectins may contribute to a protective immune response, for instance during infections, but they may also be involved in immune pathology, for instance during sterile inflammation. Main classes of lectins in innate immunity include C-type lectin receptors (CLRs), siglecs, and galectins. Due to the manifold functions of these different classes of lectins in anti-microbial defense as well as immune homeostasis, lectin targeting is a promising strategy to shape immune responses in the context of infections, autoimmunity, cancer, or vaccination.

This Research Topic provides original research and discussions focusing on aspects such as ligand recognition by lectin receptors, induced signaling pathways, and lectin-mediated effector functions during infections and inflammatory processes. It also addresses the identification and characterization of novel lectin ligands and discusses how lectin targeting can be exploited to stimulate or modulate immune responses. The contributions to this Research Topic provide in-depth insights into current research on the impact of lectins/lectin ligands on immune responses. The collection of 18 articles published in this Research Topic comprises original Research Articles, Methods and Opinion Articles, as well as Comprehensive and Mini-Reviews.

## Novel Link Between Dectin-2 and LC3-asscociated Phagocytosis of Mycobiota

It is well-known that the CLR Dectin-2 contributes to fungal recognition. Previous studies have shown an involvement of the microtubule-associated protein light-chain 3 (LC3)-associated phagocytosis (LAP) in host/fungi interactions. The study by Lamprinaki et al. describes a novel link between Dectin-2 and LAP in the host response to commensal fungi. By using defined knockout systems, the authors show a connection between Dectin-2 binding of commensal yeasts, internalization, and signaling via association with LAP.

## CLRs as Novel Markers of DC Subsets

Conventional dendritic cells (cDCs) in humans can be subdivided into two subsets, CD141^+^ DCs (cDC1) and CD1c^+^ DCs (cDC2). Heger et al. identify the CLR CLEC10A (also known as Macrophage galactose-type C-type lectin) as a specific marker for human cDC2. CLEC10A targeting using a CLEC10A-specific monoclonal antibody as well as a bivalent ligand resulted in a rapid internalization into cDC2 and increased cytokine responses upon TLR7/8 stimulation. This study highlights CLEC10A as a candidate receptor for *in vivo* antigen-targeting approaches.

## CLR Targeting for Antigen Delivery and Immune Stimulation

Targeting CLRs expressed by antigen-presenting cells is a promising strategy to enhance immune responses and to increase the efficacy of vaccines. Numerous studies have shown that the CLR DC-SIGN serves as an attractive target for the delivery of antigens into DCs; however, the mechanism of how DC-SIGN affects cross-presentation of antigens has not yet been fully elucidated. The study by Horrevorts et al. addresses the dynamics of DC-SIGN-mediated internalization, antigen processing, and cross-presentation and analyzes the impact of Toll-like receptor-4 (TLR4) on these processes. The authors conclude that the combined use of DC-SIGN and TLR4 ligands may serve as an antigen-targeting platform to boost antigen cross-presentation to CD8^+^ T cells.

Velasquez et al. use an antibody-mediated DC-SIGN targeting approach to enhance CD4^+^ T cell responses against mycobacterial antigens. Using the hSIGN mouse expressing human DC-SIGN under control of the murine CD11c promotor, they observed increased frequencies of antigen-specific IFN-γ^+^IL-2^+^TNF-α^+^ polyfunctional CD4^+^ T cells upon antigen targeting to DC-SIGN. Using a similar targeting approach for CD209a/SIGNR5 (mouse DC-SIGN), Schetters et al. show that ovalbumin (OVA)-conjugated anti-SIGNR5 antibody elicited robust antigen-specific CD4^+^ and CD8^+^ T cell responses and enhanced OVA-specific antibody responses *in vivo*. These findings highlight the utility of this CLR as a target for antigen delivery and immune stimulation.

## Identifying Novel CLR/bacteria Interactions

In order to identify novel interactions of lectin receptors with pathogens, appropriate research tools are needed. In their Methods article, Mayer et al. describe applications of CLR-Fc fusion proteins to screen for yet unknown CLR/bacteria interactions. Using *Campylobacter jejuni* as an example, they identify Dectin-1 as a candidate CLR in *C. jejuni* recognition whose functional role can now be investigated in further studies.

## Association of C-type Lectin With Pulmonary Tuberculosis

CLRs have been implicated in the recognition of mycobacteria and the induction of anti-mycobacterial immunity (see the review article by Wagener et al. in this Research Topic). Klassert et al. used an AmpliSeq-based approach to screen main CLR gene clusters and CLR pathway-related genes for single nucleotide polymorphisms (SNPs) associated with pulmonary tuberculosis in an Indian population. One SNP in the gene encoding for the Mannan-binding lectin serine protease 1 (MASP1) was found to be significantly associated with pulmonary tuberculosis in this population, thus suggesting an involvement of the lectin pathway of the complement system in tuberculosis pathogenesis.

## Role of Siglecs and Galectins in Immune Modulation

While most of the articles included in this Research Topic deal with CLRs, the two other main classes of lectins in innate immunity, siglecs, and galectins, must not be underestimated. Siglecs represent a lectin superfamily that is widely expressed by immune cell subsets and characterized by binding to sialic acid residues. The study by Nagala et al. investigates a potential role of Siglec-E as a negative regulator of TLR4-mediated endocytosis and signaling as proposed in previous studies. However, while Siglec-E induction by bacterial lipopolysaccharide (LPS) modulated the phenotype of macrophages, their study does not support a significant role for Siglec-E in TLR4-mediated endocytosis and signaling functions.

Galectins share a common structural fold and exhibit a preference for *N*-acetyllactosamine-containing glycoconjugates. In their Perspective article, Sundblad et al. discuss findings on galectin functions in intestinal inflammation. Their data indicate that galectins represent active players in the intestinal mucosa to preserve immune and epithelial homeostasis. Thus, galectins may serve as promising biomarkers and therapeutic targets during severe mucosal inflammation.

## CLRs in Infection, Inflammation, and Autoimmunity

The Research Topic is complemented by comprehensive review and opinion articles focusing on the functions of CLRs in infection, inflammation and autoimmunity. In their review article, Goyal et al. describe CLR interaction with human pathogenic fungi. The authors summarize known functions of CLRs, such as Dectin-1, Dectin-2, Mincle, Mannose receptor (MR), and DC-SIGN, in fungal recognition and highlight their relevance in orchestrating antifungal responses. Besides their role in antifungal immune responses, CLRs are also involved in viral recognition. How CLRs contribute to antiviral immunity on the one hand, but may also be exploited by viruses to escape immune responses on the other hand, is the focus of the review article by Bermejo-Jambrina et al. The authors provide an overview of known interactions between viruses and CLRs, including the impact of CLR engagement on virus internalization, transmission, and cross-presentation of viral antigens. In addition, CLRs contribute to innate and adaptive immune responses during viral infection, such as type-I interferon responses or T helper cell polarization. Cross-talk mechanisms between CLRs and complement receptors in opsonization and virus internalization are discussed in this review as well. Wagener et al. review the relevance of the Dectin-1/Syk/CARD9 signaling axis in anti-mycobacterial immunity. The authors specify Dectin-1 functions in recognition of mycobacteria and discuss recent findings on the crucial role of Syk signaling and the adaptor protein CARD9 in anti-mycobacterial immunity. While a distinct mycobacterial PAMP recognized by Dectin-1 still remains to be identified, this review highlights the relevance of the Dectin-1 pathway in mounting an immune response to *Mycobacterium tuberculosis*, which may lead to potential applications in tuberculosis vaccine adjuvant development.

CLRs not only play an important role in the recognition of pathogens, but may also sense DAMPs and contribute to sterile inflammation and autoimmunity. Chiffoleau reviews this essential role of CLRs as driving players of sterile inflammation and highlights their relevance for autoimmune diseases, allergy, or cancer. Specific aspects of how myeloid CLRs sense their cognate ligands and crosstalk with heterologous receptors are detailed by del Fresno et al. Their review article deals with the diversity of signaling modules in CLRs. Signaling may require CLR multimerization; the relative affinity or avidity of CLR ligands may determine whether an activatory or inhibitory signal is finally conveyed into the CLR-expressing cell. In conclusion, further research is needed to understand how different signaling pathways triggered by CLRs and heterologous receptors act in concert.

Two articles in this Research Topic focus on CLRs in autoimmune diseases. While Hadebe et al. review current knowledge on the role of CLRs recognizing fungus-derived and other allergens in asthma, the opinion article contributed by te Velde discusses clues for a role of the CLR Mincle in Crohn's disease. The Research Topic is rounded by two review articles on structural aspects of ligand recognition by the CLR Mincle and the identification and characterization of novel Mincle ligands. Williams discusses the structural basis of Mincle recognition of lipidic ligands including sterols, glucose- and glycerol-based glycolipids, and glycosyl diglycerides. This review also details how variations of Mincle ligands have provided insights into structure-activity relationships and may help to identify novel Mincle ligands. Braganza et al. focus on the chemical nature of known Mincle ligands and the rational synthesis of analogs to screen for potent Mincle agonists. Interestingly, subtle changes to functional groups in the lipid backbone of Mincle ligands were found to markedly affect their immune stimulatory properties. In conclusion, Mincle agonists display promising potential as vaccine adjuvants and immunotherapeutics.

## Conclusions

Mounting evidence has demonstrated that lectin receptors play crucial roles in innate immunity. This Research Topic provides numerous examples of how lectins are involved in immune responses during infections or inflammatory processes. It also highlights the utility of lectin targeting for antigen delivery to enhance vaccine efficacy. Future studies will unravel the potential of lectins as therapeutic targets during infections, autoimmune diseases, or cancer.

## Author Contributions

All authors listed have made a substantial, direct and intellectual contribution to the work, and approved it for publication.

### Conflict of Interest

The authors declare that the research was conducted in the absence of any commercial or financial relationships that could be construed as a potential conflict of interest.

